# Human TRAV1-2-negative MR1-restricted T cells detect *S. pyogenes* and alternatives to MAIT riboflavin-based antigens

**DOI:** 10.1038/ncomms12506

**Published:** 2016-08-16

**Authors:** Erin W. Meermeier, Bruno F. Laugel, Andrew K. Sewell, Alexandra J. Corbett, Jamie Rossjohn, James McCluskey, Melanie J. Harriff, Tamera Franks, Marielle C. Gold, David M. Lewinsohn

**Affiliations:** 1 Department of Molecular Microbiology and Immunology, Oregon Health and Science University, Portland, Oregon 97239 USA; 2 Institute of Infection and Immunity, Henry Wellcome Research Institute, Cardiff University School of Medicine, Cardiff CF14 4XN, UK; 3 Department of Microbiology and Immunology, Peter Doherty Institute for Infection and Immunity, University of Melbourne, Parkville, Victoria 3010, Australia; 4 Department of Biochemistry and Molecular Biology, School of Biomedical Sciences, Monash University, Clayton, Victoria 3800, Australia; 5 ARC Centre of Excellence in Advanced Molecular Imaging, Monash University, Clayton, Victoria 3800, Australia; 6 Department of Pulmonary and Critical Care Medicine, Oregon Health and Science University, Portland, Oregon 97239, USA; 7 Department of Research, VA Portland Health Care Center, Portland, Oregon 97239, USA

## Abstract

Mucosal-associated invariant T (MAIT) cells are thought to detect microbial antigens presented by the HLA-Ib molecule MR1 through the exclusive use of a TRAV1-2-containing TCRα. Here we use MR1 tetramer staining and *ex vivo* analysis with mycobacteria-infected MR1-deficient cells to demonstrate the presence of functional human MR1-restricted T cells that lack TRAV1-2. We characterize an MR1-restricted clone that expresses the TRAV12-2 TCRα, which lacks residues previously shown to be critical for MR1-antigen recognition. In contrast to TRAV1-2^+^ MAIT cells, this TRAV12-2-expressing clone displays a distinct pattern of microbial recognition by detecting infection with the riboflavin auxotroph *Streptococcus pyogenes*. As known MAIT antigens are derived from riboflavin metabolites, this suggests that TRAV12-2^+^ clone recognizes unique antigens. Thus, MR1-restricted T cells can discriminate between microbes in a TCR-dependent manner. We postulate that additional MR1-restricted T-cell subsets may play a unique role in defence against infection by broadening the recognition of microbial metabolites.

## Abstract (web summary)

Mucosal-associated invariant T (MAIT) cells sense riboflavin biosynthetic intermediate antigens with a semi-invariant MR1-restricted T-cell receptor (TCR). Here the authors identify a new MR1-restricted TCR that senses cells infected with *S. pyogenes*, a bacteria unable to biosynthesize riboflavin.

## Graphical abstract

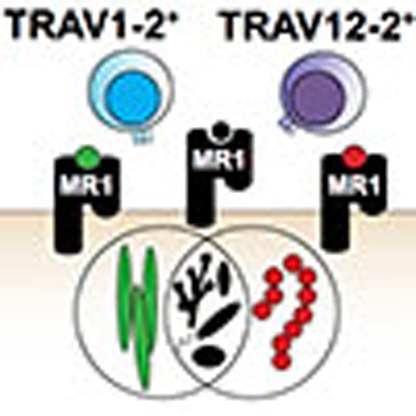

Human mucosal-associated invariant T (MAIT) cells have been described as an abundant population of αβ-T-cell antigen receptor (TCR) T cells that display antimicrobial Th1-like cytotoxic capacity upon detection of a range of microbial infections^1^,^2^,^3^. By definition, MAIT cells express a semi-invariant TCR that engages antigenic ligands presented by the HLA-Ib major histocompatibility complex (MHC)-related protein I (MR1). MR1 has been shown to present small compounds derived from folic acid and riboflavin biosynthesis, the latter of which can activate MAIT cells^4^,^5^,^6^. In healthy humans, MAIT cells account for 1–10% of T cells in peripheral blood. They are also abundant in the liver and in a number of mucosal tissues^1^,^7^,^8^,^9^,^10^,^11^. Thymic selection and peripheral expansion of MAIT cells depend on MR1 (refs 11, 12). Furthermore, MAIT cells with effector function have been found in the thymus, a finding that has been used to support their definition as innate-like^13^. While the role of MR1 and MAIT cells in human immunity is unclear, mouse studies have demonstrated their role in protection against bacterial infections including *Klebsiella pneumoniae*, *Mycobacterium bovis* Bacillus Calmette–Guérin (BCG) and *Francisella tularensis* live vaccine strain (LVS)^14^,^15^,^16^.

MR1 is an HLA-Ib MHC class I molecule thought to be highly conserved in mammalian evolution^17^. MR1 can bind vitamin B-based precursors derived from folic acid (vitamin B9) and riboflavin (vitamin B2) biosynthesis that share a common pterin ring structure^5^. So far, only those from the riboflavin synthetic pathway have been shown to stimulate MAIT cells. These stimulating ligands can be derived from either pyrimidine-based early intermediates in riboflavin synthesis (5-A-RU) that form adducts with other small metabolites (for example, 5-OP-RU) or the direct lumazine precursors of riboflavin (for example, ribityllumazine (RL)-6,7-diMe)^4^,^5^. Because riboflavin synthesis does not occur in humans, riboflavin metabolites presented in the context of MR1 have been suggested to be pathogen-associated molecular patterns. However, evidence supports the existence of additional MR1 ligands. For example, structural analysis suggests that plasticity in the MR1-binding groove could accommodate a range of different ligands^4^,^18^,^19^,^20^,^21^,^22^. As the pterin ring occurs commonly in the environment, it is feasible that other microbial or host molecules with common chemotypic properties could bind to MR1 and function as antigens for MR1-restricted T cells.

Although MAIT cells specifically recognize infection by pathogens with the capacity to synthesize riboflavin^1^,^3^, whether microbe-specific MR1 ligands exist is unknown. We previously evaluated the *ex vivo* human TCR repertoire of MAIT cells responsive to three riboflavin-synthesizing microbes^23^, finding that distinct MAIT TCR usage was associated with microbe-selective responses within and across individuals. These data support the hypothesis that MR1 can present discrete microbial ligands, and that this presentation is in turn associated with selective clonal expansion of MAIT cells. However, it is not known whether each microbe synthesizes the same repertoire of riboflavin metabolites, but at varying proportions, or whether there are unique ligands.

The nature of the diversity in MR1 ligand repertoire suggests an accordingly diverse MAIT TCR repertoire to mediate ligand recognition. Human MAIT TCRα chains have been described as being invariant, comprising *TRAV1-2/TRAJ12, 20, 33* genes paired with a limited array of TCR β-chains^1^,^11^,^13^,^24^,^25^. However, other studies have identified greater TCR heterogeneity through more diverse TCRα and TCRβ chain usage^10^,^23^,^26^,^27^,^28^. Gherardin *et al.*^28^ described TRAV1-2-negative TCRs that bind selectively to MR1 tetramers loaded with 5-OP-RU (riboflavin metabolite), or 6FP/acetyl-6FP (folate derivative), or both. These TRAV1-2-negative TCRs represent unprecedented diverse TRAV and TRBV usage by MR1-restricted T cells. These findings suggest that MR1-restricted T cells could use diverse TCRs to recognize microbial infection; therefore, the full repertoire of TCRs that can be used by MR1-restricted T cells is unknown.

Here we describe microbe-reactive MR1-restricted T cells that do not express TRAV1-2. Functional analysis reveals that these cells, although less prevalent than those that express TRAV1-2, can be found in PBMC from all individuals. Among MR1-Ag tetramer-positive cells, 1–4% are TRAV1-2-negative. T-cell cloning confirms the usage of an alternative TCRα chain, TRAV12-2, by an MR1-restricted T-cell clone from one donor. In comparison with previously described TRAV1-2^+^ MAIT cells, this T-cell clone displays a unique pattern of ligand and microbial selectivity. Most notably, the TRAV12-2 T-cell clone could detect infection with *Streptococcus pyogenes* in a TCR-dependent manner, a microbe that is not capable of synthesizing riboflavin. These data, then, provide direct evidence of the ability of MR1 to present a diverse array of ligands, which in turn is associated with selective TCR usage. Finally, our findings challenge the current paradigm of sole usage of TRAV1-2 in conjunction with the recognition of riboflavin metabolites being the defining feature of MR1-restricted T cells.

## Results

### Enumeration of functional TRAV1-2^−^ MR1-restricted T cells

**Figure 1:**
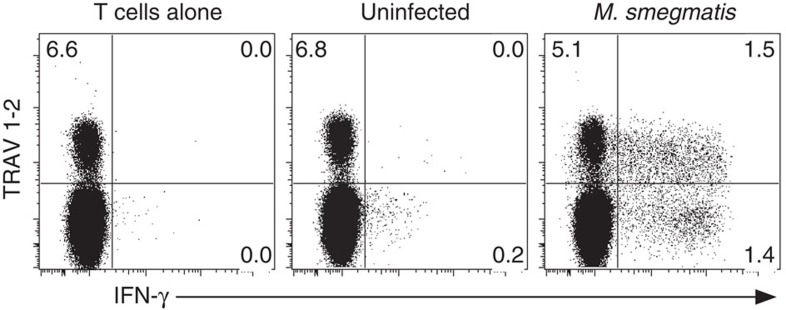
*M. smegmatis* infection elicits a response from MAIT cells and TRAV1-2^−^ HLA-Ib-restricted CD8^+^ T cells. PBMC-derived CD8^+^ T cells from adult donor (D462) were stimulated *ex vivo* by A549 cells infected with *M. smegmatis* to identify mycobacteria-reactive HLA-Ib T cells. Functional MAIT cells were identified by production of IFN-γ and expression of TRAV1-2 TCR. Numbers in quadrants indicate the % of live CD3^+^ cells. Experiments were performed three independent times with similar results. Representative results are shown.

MAIT cells can detect a wide range of bacteria and fungi through recognition of riboflavin metabolites presented by the HLA-Ib molecule MR1. In this context, we sought to explore the relative contribution of MR1 to the entire HLA-Ib-restricted CD8^+^ T-cell response to microbial infection. In order to quantify and characterize these responses directly *ex vivo*, we have developed a functional *ex vivo* assay that relies upon cytokine production by CD8^+^ T cells in response to microbial infection of HLA-mismatched A549 cells^1^. The flow cytometry gating scheme used to analyse this response is shown in Supplementary Fig. 1. Using this approach, we have consistently been able to enumerate MAIT cells (TRAV1-2^+^) responsive to a number of microbes such as Mtb^1^,^13^,^23^, *Candida albicans* and *Salmonella typhimurium* infections^23^. However, we also consistently observed TRAV1-2-negative cells reactive to these same microbes. For example, nearly 50% of the CD8^+^ HLA-Ib response to *M. smegmatis* (*M. smegmatis*) was TRAV1-2-negative in a representative donor, D462 (Fig. 1).

**Figure 2:**
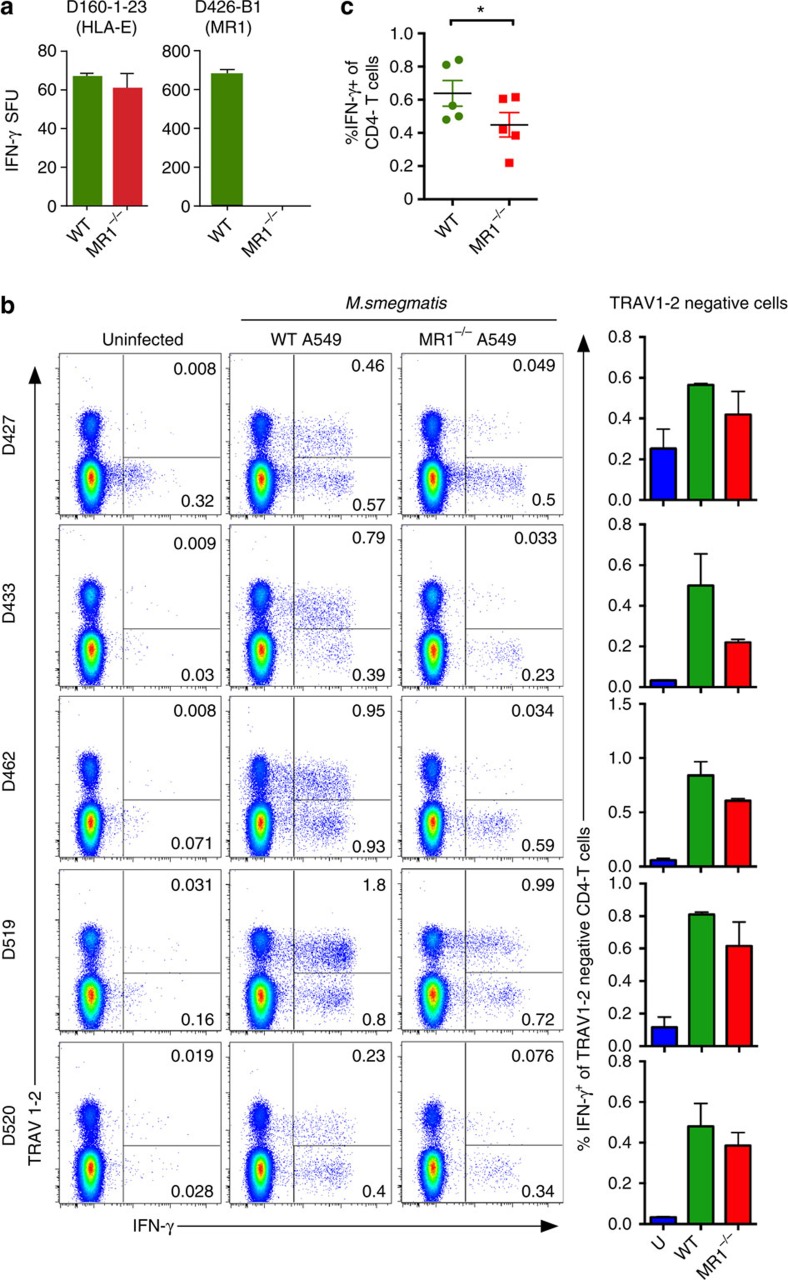
MR1-restricted microbial-reactive CD8^+^ T cells from blood do not exclusively express TRAV1-2. (**a**) IFN-γ production by T-cell clones D160-1-23 (restricted by HLA-E) and D426-B1 (restricted by MR1) in response to mycobacteria-infected WT and MR1^−/−^ A549 cell line. (**b**) Positively selected CD8^+^ T cells from PBMC were tested for *ex vivo* IFN-γ responses to *M. smegmatis*-infected WT or MR1^−/−^ A549 cell line. Events are gated on live CD3^+^CD4^−^ cells. IFN-γ and TRAV1-2 expressions are shown on the *x* and *y* axes, respectively. To the right is a summary of the TRAV1-2-negative response from each donor across experiments. (**c**) Frequency of IFN-γ^+^ CD4^−^ cells from each donor (represented by one dot) when stimulated by *M. smegmatis*-infected WT or MR1^−\−^ A549s, *n*=5 biological replicates, with *n*=3 technical replicates. Statistical significance of difference between groups was determined using the nonparametric Mann–Whitney *U*-test. Error bars are the s.e.m. of triplicates in **a**,**b**. Experiments in this figure were performed at least twice with similar results. Representative results are shown. *P value>0.05 was considered significant.

To address the hypothesis that TRAV1-2-negative cells were MR1-restricted, we generated an MR1-knockout A549 cell line^29^. Briefly, CRISPR/Cas9 technology was used to generate a single base pair deletion and a 125-bp deletion separately on each allele to ablate expression of MR1. To confirm the absence of MR1 in a functional assay, the wild-type (WT) and MR1^−/−^ cell lines were infected with mycobacteria and T-cell responses evaluated by interferon (IFN)-γ ELISPOT. As shown in Fig. 2a, activation of the TRAV1-2^+^ MR1-restricted clone (D426-B1 (ref. 23)) was ablated, while activation of the HLA-E-restricted clone (D160-1-23) was unaffected, indicating that lack of MR1 did not affect infectivity or a separate antigen-presentation pathway.

To establish the prevalence of MR1-restricted T-cell responses *ex vivo*, WT and MR1^−/−^ A549 cells were infected with *M. smegmatis* and used as stimulators for CD8^+^ T cells isolated from PBMC of five healthy individuals (Fig. 2b). Intracellular IFN-γ production was assessed using flow cytometry. As expected, TRAV1-2^+^ cells produced IFN-γ in response to *M. smegmatis*-infected WT A549 cells. Furthermore, the majority of the response was MR1-dependent (mean 85.21% MR1-restricted, range 45.3–97.22, *n*=5, tested in duplicate). Each donor also had a proportion of TRAV1-2-negative cells whose production of IFN-γ was MR1-dependent (mean 25.83% MR1-restricted, range 10–41.03, *n*=5, tested in duplicate; Fig. 2b). The average of the cytokine responses by the TRAV1-2-negative population for each donor across experiments was plotted in Fig. 2b (right panel). In order to confirm the presence of TRAV1-2-negative *M. smegmatis*-reactive T cells, we repeated this assay using PBMC that were fluorescence-activated cell sorting (FACS)-selected on CD8 but depleted of TRAV1-2^+^ cells. Then, we stimulated the T cells with WT (MR1-positive) infected A549 cells. From all donors we observed a detectable IFN-γ-producing population. Therefore, we conclude that not all microbe-reactive MR1-restricted T cells are TRAV1-2^+^.

### Cloning TRAV1-2-negative MR1-restricted T cells

**Figure 3:**
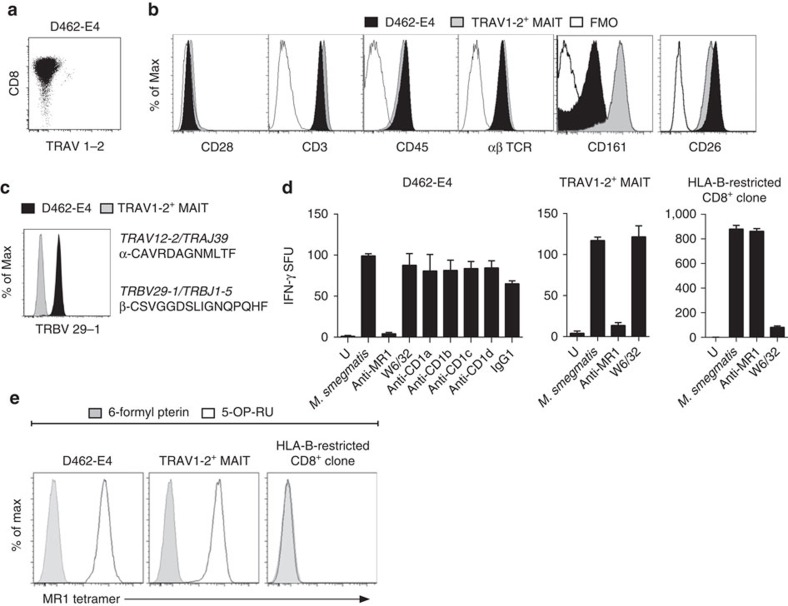
Isolation of an MR1-restricted T-cell clone that reacts to bacteria independently of the TRAV1-2 TCR. Generation of a CD8^+^ T-cell line and then T-cell clone, D462-E4, through FACS sorting on CD8^+^, γδ TCR^−^, TRAV1-2-negative T cells from donor in (Fig. 1) and co-culturing for 7 days with *M. smegmatis*-infected autologous DCs. The line was stained with CFSE and co-cultured for 7 days with *M. smegmatis*-infected autologous macrophages. (**a**) Staining of TRAV1-2 and co-receptor CD8 on T-cell clone D462-E4. (**b**) Comparison of T-cell surface marker expression on TRAV1-2-negative clone D462-E4 (black) and TRAV1-2^+^ MAIT clone D426-G11 (grey) and (**c**) of TRBV 29-1 on D462-E4 and D481-A9 TRAV1-2^+^ MAIT clone. TCR αβ CDR3 sequences and gene names of D462-E4 are shown, right. (**d**) BEAS2B cell line infected with mycobacteria and tested for their ability to stimulate T-cell clones in the presence of blocking antibodies or isotype by IFN-γ ELISPOT assays, D462-E4, D481-F12 (TRAV1-2^+^ MAIT) and D466-A10 (HLA-B45). (**e**) Flow cytometry of clones with MR1 tetramer loaded with 6-formyl pterin (grey) or 5-OP-RU (clear). D462-E4, D481-A9 MAIT clone and D466-A10 HLA-B45-restricted T-cell clone. Error bars represent the s.e.m. of triplicates. Experiments were performed at least two times with similar results. Representative results are shown.

To further characterize TRAV1-2-negative MR1-restricted CD8^+^ T cells in each of the five donors, we used *M. smegmatis*-infected antigen-presenting cell (APC) to generate CD8^+^ TRAV1-2-negative T-cell lines that were both reactive to *M. smegmatis* and whose activation was blocked by the addition of MR1 antibody. Subsequent cloning of the T-cell line from one donor, D462, using α-CD3 stimulation was used to establish the T-cell clone, D462-E4. As shown in Fig. 3a, D462-E4 was characterized by the uniform expression of CD8α and the absence of TRAV1-2. In comparison with TRAV1-2^+^ MAIT cell clones, D462-E4 expressed equivalent levels of TCR and co-stimulatory receptors (Fig. 3b). This T-cell clone also expressed CD26 (ref. 30) but did not express CD161 (ref. 7), although CD161 may have been downregulated as the result of expansion with anti-CD3 (refs 30, 31; Fig. 3b). TCR sequence analysis of D462-E4 demonstrated the unique expression of *TRAV12-2/TRAJ39* TCRα and *TRBV29-1/TRBJ1-5* TCRβ (Fig. 3c and Supplementary Table 1). The expression of the TCRβ chain was confirmed by antibody staining, which showed staining on D462-E4 but not by MAIT T-cell clone, D481-A9, that expresses TRBV20-1^1^. We confirmed that the clone was *M. smegmatis*-reactive using infected epithelial cells, and restricted with MR1 using antibody blockade (Fig. 3d). The D462-E4 clone retained the ability to recognize *M. smegmatis*-infected cells in a manner that was blocked by α-MR1, but not by the pan-HLA I antibody W6/32, nor by any of the CD1-blocking antibodies. These results are similar to the pattern seen by the known MR1-restricted T-cell clones (D481-F12, Fig. 3d). The clonality and MR1 reactivity of D462-E4 was confirmed by staining with the MR1-Ag tetramer loaded with the MAIT-activating ligand, 5-OP-RU (Fig. 3e). Notably, clone D462-E4 bound the MR1-Ag tetramer with the same level of intensity as the TRAV1-2^+^ MAIT cell clone D426-B1 (ref. 1), while the HLA-B45-restricted clone D466-D6 (ref. 32) did not bind the tetramer. In contrast, neither MR1-restricted clone bound the MR1 tetramer loaded with the MAIT cell inhibitor, 6-formyl pterin (6FP). Collectively, isolation of clone D462-E4 confirms the presence of microbe-reactive MR1-restricted CD8^+^ T cells that detect infection using an atypical MAIT TCR TRAV12-2.

### Differential antigen recognition by MR1-restricted T cells

**Figure 4:**
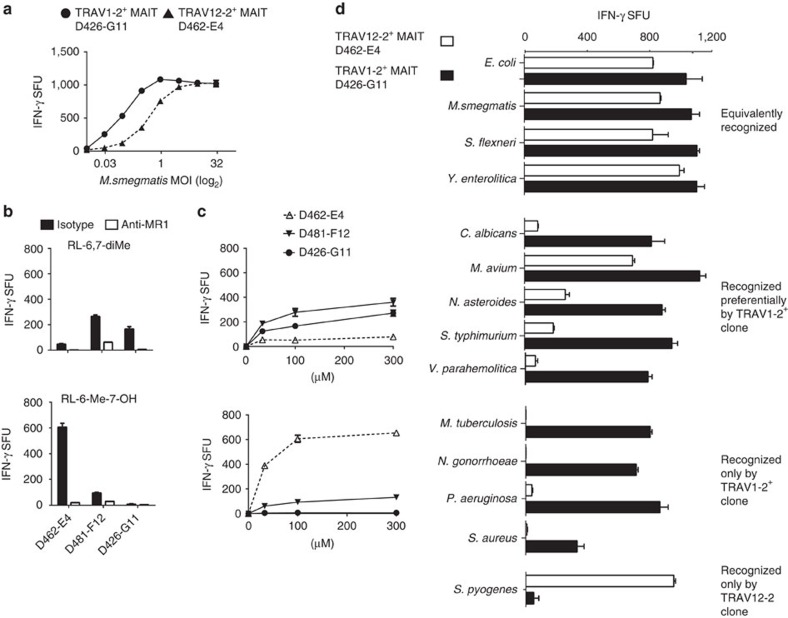
T-cell clone D462-E4 displays ligand and microbial infection selectivity. (**a**) DCs were infected with *M. smegmatis* at a range of MOI (*x* axis) and then co-incubated with indicated MR1-restricted T-cell clones. (**b**) A549 cells were pulsed for 2 h with 100 μM RL-6,7-diMe (top) or RL-6-Me-OH (bottom) and tested for their ability to stimulate MR1-restricted T-cell clones in the presence of α-MR1 or isotype antibody by IFN-γ ELISPOT. (**c**) A549 cells were pulsed for 2 h with a range of concentrations of RL-6,7-diMe (top) or RL-6-Me-OH (bottom) and tested for their ability to stimulate MR1-restricted T-cell clones by IFN-γ ELISPOT. (**d**) DCs were infected at optimized MOIs with pathogens listed on the *y* axis, except for *Yersinia* and *Shigella* where the A549 cell line was used, and then co-incubated overnight with the indicated MR1-restricted T-cell clones. IFN-γ production was quantified by ELISPOT. Detection of less than 25 spot-forming unit per well was considered no response by the T-cell clone. Results are grouped by comparative response by MR1-restricted T-cell clones. Pattern of recognition was maintained over a range of MOI. Error bars are the s.e.m. of triplicates. Assays were performed twice, with similar results. Representative results are shown.

Because we had isolated D462-E4 from an *M. smegmatis*-reactive T-cell line, we wanted to compare the *M. smegmatis* reactivity of TRAV1-2^+^ and TRAV12-2^+^ T-cell clones over a range of MOI. In Fig. 4a, we tested clone D426-G11 and D462-E4 in an IFNγ ELISPOT for their reactivity to infected dendritic cells (DCs). Although both clones recognized the infection, our data show a higher potency of antigen dose in regards to the TRAV1-2^+^ clone (D426-G11), while both clones displayed similar maximal efficacy (cytokine release). This result suggested that the TRAV12-2 TCR either had lower TCR avidity or could recognize fewer MR1 ligands from the infected cell. Given prior evidence of MR1 ligand discrimination^1^,^4^,^20^,^23^,^28^, we tested whether the TRAV12-2 TCR had different ligand selectivity in comparison with the TRAV1-2 MAIT TCR. To define the MR1 ligands recognized by the TRAV12-2^+^ TCR, we first tested D462-E4 for its ability to recognize the A549 cell line loaded with MAIT RL antigens RL-6,7-diMe and RL-6-Me-7-OH (ref. 5) in the presence or absence of MR1 blockade. As shown in Fig. 4b, D462-E4 detected both antigens in an MR1-dependent manner. However, D462-E4 was preferentially stimulated by RL-6-Me-7-OH. In contrast, two previously characterized MR1-restricted clones D481-F12 and D426-G11 (refs 1, 23) were preferentially stimulated by the RL-6,7-diMe antigen. To better understand whether this differential response was due to TCR avidity, T-cell activation was tested over a range of antigen concentrations. We found that the differential responses by D462-E4 compared with the other MR1-restricted T-cell clones were maintained over a wide range of concentrations (Fig. 4c). In these experiments, we observed similar antigen potency (antigen concentration of half maximum response) yet different maximal efficacy (cytokine release) in response to the two-RL antigens between the three MR1-restricted T-cell clones. These responses may be indicative of different levels of antigen cross-reactivity between these TCRs. Therefore, we concluded that D462-E4 displayed ligand discrimination between MAIT RL antigens.

### Differential recognition of microbes by MR1-restricted T cells

To establish the repertoire of microbes recognized by D462-E4, a diverse array of microbes was tested. DCs and epithelial cells were infected with microbes at optimized MOI and used to compare the ability of T-cell clones D462-E4 (TRAV12-2) and D426-G11 (TRAV1-2) to recognize these targets (Fig. 4d). We observed four patterns of responses from the MAIT T-cell clones: (1) microbes recognized equivalently by both T-cell clones (*M. smegmatis, E. coli, Yersinia enterolitica* and *Shigella flexneri*), (2) microbes preferentially recognized by the TRAV1-2^+^ T-cell clone (*M. avium, S. typhimurium, C. albicans*, *Nocardia asteroides* and *Vibrio parahemolytica*), (3) microbes only detected by the TRAV1-2^+^ T-cell clone (*M. tuberculosis, Staphylococcus aureus, Pseudomonas aeruginosa* and *Neisseria gonorrhoeae*) and (4) microbes only recognized by TRAV12-2^+^ T-cell clone (*S. pyogenes;* Fig. 4d). Neither clone responded to the riboflavin auxotroph *Enterococcus faecalis.* Thus, the TRAV1-2^+^ MAIT clone, D426-G11, only responded to microbes with the capacity to produce riboflavin. While the D462-E4 clone was also able to recognize many of these microbes (Fig. 4d), it was distinguishable in its ability to respond to infection with *S. pyogenes.*

**Figure 5:**
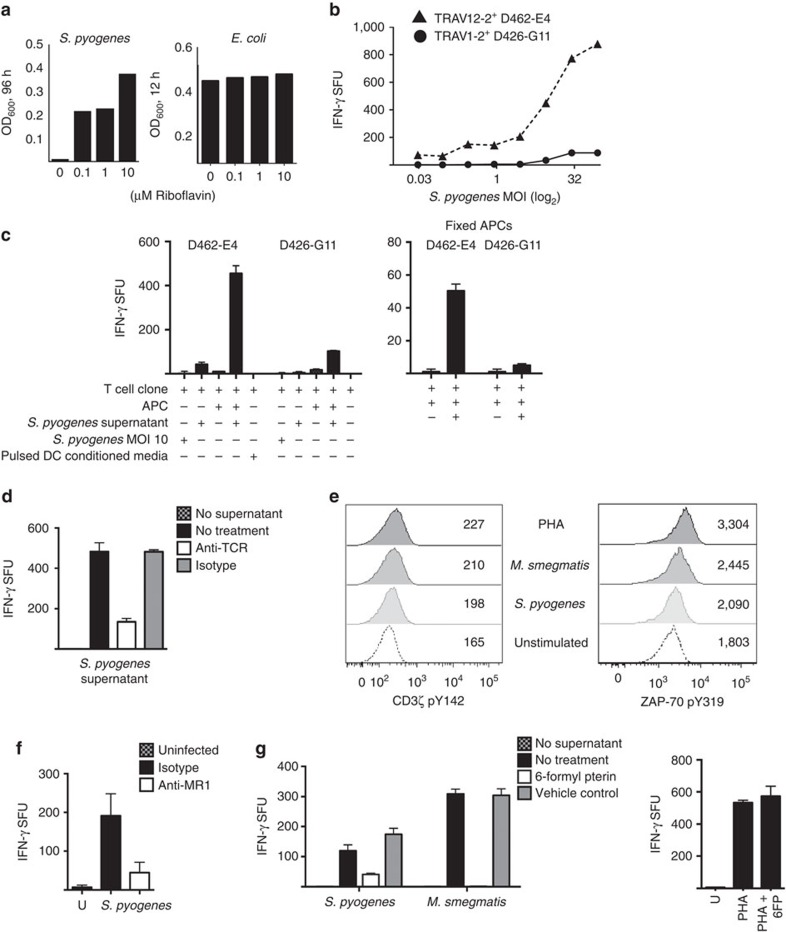
Selective recognition of *S. pyogenes* by a TRAV1-2-negative T-cell clone D462-E4. (**a**) *S. pyogenes* or *E. coli* was cultured in minimal broth without riboflavin or with a titration of riboflavin over the course of 96 h (*S. pyogenes*) or 12 h (*E. coli*), and growth was monitored by optical density readings. (**b**) DCs were infected with *S. pyogenes* at a range of MOI (*x* axis) and then co-incubated with indicated MR1-restricted T-cell clones. (**c**) T-cell clones D462-E4 and D426-G11 were incubated with (left to right) *S. pyogenes* (MOI=10), *S. pyogenes* culture supernatant (SN) 30 μl, unloaded DCs, DCs with *S. pyogenes* SN or pulsed DC-conditioned media overnight. On the right, D462-E4 and D426-G11 were incubated with paraformaldehyde-fixed DCs that had been pulsed with *S. pyogenes* SN. IFN-γ production was quantified by ELISPOT. (**d**) T-cell clone D462-E4 was blocked with anti-pan TCR αβ, isotype control or not treated and then co-incubated with DCs loaded with *S. pyogenes* supernatant (30 μl). IFN-γ production was quantified by ELISPOT. (**e**) Phosphorylation of the CD3-ζ chain of the TCR/CD3 complex at tyrosine 142 or ZAP-70 at tyrosine 319 was quantified by flow cytometry after T-cell clone D462-E4 was co-incubated with DCs infected with *S. pyogenes or M.smeg* MOI=10, PHA (20 μg ml^−1^) or left untreated (unstimulated condition). Numbers on the overlay indicate the geometric mean fluorescence intensity of at least 30,000 clones. (**f**) DCs were infected with *S. pyogenes* at MOI=3, blocked with anti-MR1 or isotype control (10 μg ml^−1^) and then co-incubated with D462-E4 T-cell clone. IFN-γ production was quantified by ELISPOT. (**g**) DCs were either blocked with 6-formyl pterin (50 μg ml^−1^) or 0.01 M NaOH vehicle control or nothing, and then loaded with *S. pyogenes or M.smeg* supernatant (15 μl) or PHA at 10 μg ml^−1^. The DCs were then used to stimulate T-cell clone D462-E4 and IFN-γ production was quantified by ELISPOT. Error bars represent the s.e.m. of at least duplicates. Assays were performed three times, with similar results. Representative results are shown.

*S. pyogenes* is a bacterium that does not express riboflavin synthesis Rib enzymes^33^,^34^,^35^ *ribA, ribB, ribD, ribH* and *ribE* (*E. coli* operon nomenclature). Initially, we confirmed that *S. pyogenes* was auxotrophic for this vitamin (Fig. 5a). To demonstrate the selectivity of the TRAV12-2 T-cell clone for *S. pyogenes*, clones were tested over a broad range of MOIs. The TRAV12-2^+^ T-cell clone responded over a range of *S. pyogenes* MOI in DCs (Fig. 5b), while the TRAV1-2^+^ clone did not respond to this infection. To exclude a nonspecific effect on the TRAV12-2 clone, cells were incubated with *S. pyogenes* or filtered culture supernatant without an APC. Here no activation was observed (Fig. 5c). In order to establish whether the T-cell clone was activated by soluble factors from the APC, we used two approaches. First, we tested whether conditioned media from DCs pulsed with *S. pyogenes* culture supernatant could activate D462-E4. Here no activation was observed (Fig. 5c). Second, we tested whether pulsed fixed DCs would maintain the same pattern of eliciting T-cell clone activation as unfixed pulsed DCs (Fig. 5c). In this experiment, DCs that had been pulsed with *S. pyogenes* supernatant overnight and then fixed were still able to activate T-cell clone D462-E4, suggesting that the response depends upon antigen presentation and not soluble factors. We next sought to determine whether the response to *S. pyogenes* was dependent upon the TCR. As shown in Fig. 5d the response to *S. pyogenes* could be blocked using the pan αβ-TCR non-activating antibody but not an isotype control. Furthermore, increased phosphorylation of CD3ζ the TCR–CD3 complex (the primary intracellular signal-transducing subunit^36^) was observed in D462-E4 following incubation with DCs infected with *M. smegmatis* or *S. pyogenes* (Fig. 5e). ZAP-70 is a tyrosine kinase that, upon TCR stimulation, is recruited to the TCR–CD3 complex by phosphorylation of the ITAMs of CD3ζ (ref. 37). After TCR engagement, the tyrosine residues Y315 and Y319 of ZAP-70 are phosphorylated. In order to provide further evidence of TCR stimulation upon recognition of bacterially infected DCs, we also compared the level of ZAP-70 pY319 between treatments. Here we also observed increased phosphorylation of this key residue of ZAP-70 in D462-E4 following incubation with DCs (Fig. 5e). To determine whether the recognition of *S. pyogenes* was dependent on MR1, two approaches were employed. First, we tested whether the clone's response to *S. pyogenes* could be blocked with antibody to MR1 (Fig. 5f). Here recognition was efficiently inhibited by addition of anti-MR1 but not isotype control. Second, we employed the MR1 antagonist, 6FP (Fig. 5g). The response to *S. pyogenes*, but not mitogen Phytohaemagglutinin (PHA), was also blocked by addition of 6FP over the vehicle control. Thus, clone D462-E4 detects both RLs MAIT antigens and an unidentified streptococcal-derived antigen in an MR1 and TCR-dependent manner. This unique detection pattern of infection and ligand recognition by TRAV12-2^+^ D462-E4 compared with TRAV1-2^+^ MAIT cells indicates a greater diversity in microbial MR1 ligands.

### Tetramer staining of TRAV1-2^−^ MR1-restricted T cells

**Figure 6:**
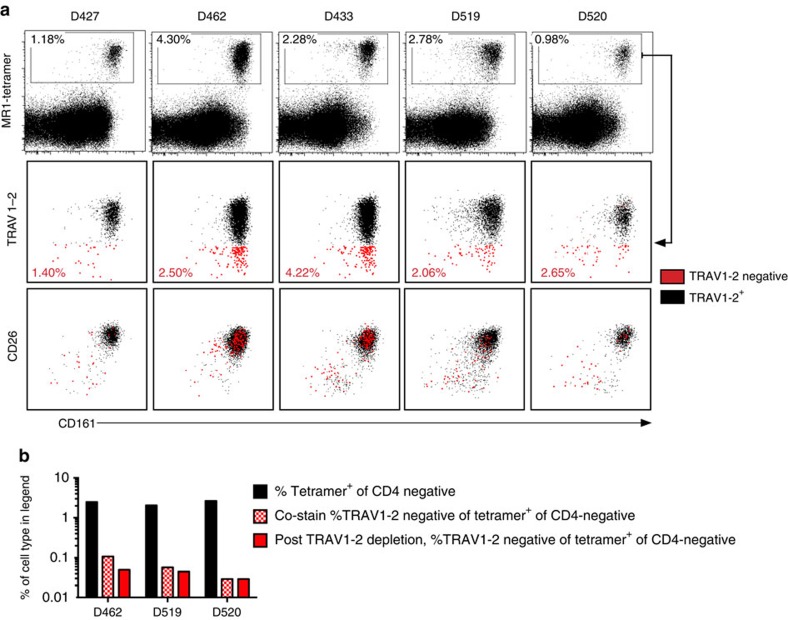
MR1-Ag tetramer^+^ CD8^+^ T cells from peripheral blood do not exclusively express TRAV1-2. PBMCs were stained with antibodies to CD3, CD4, CD8, TRAV1-2, CD26, CD161, viability stain and human MR1-Ag (5-OP-RU) tetramer. All plots are gated on live, CD3^+^, CD4-negative cells. The *x* axis represents CD161 expression, and the *y* axis represents MR1 tetramer (top row), TRAV1-2 (middle row) and CD26 (bottom row). MR1 tetramer^+^ cells were gated as indicated by the gate in the top row. MR1 tetramer^+^ cells were subgated based on TRAV1-2 expression where TRAV1-2-negative events and their frequencies are shown in red. The second and third rows of plots are overlays of TRAV1-2^+^ in black and TRAV1-2-negative in red. (**b**) PBMCs were depleted of TRAV1-2^+^ cells by FACS and then stained with MR1-Ag tetramer and T-cell lineage markers (red bars). The black bars and red-patterned bars indicate frequencies presented in (**a**) for comparison. This experiment was performed once and (**a**) was performed twice with similar results. Representative results are shown.

To establish the prevalence of TRAV1-2-negative MR1-restricted T cells across healthy individuals, we first stained PBMC with the MR1-Ag (5-OP-RU) tetramer, followed by sequential staining with antibodies to MAIT-associated surface markers (Supplementary Fig. 2). As human MAIT cells have been defined as either CD8^+^ or CD8^−^CD4^−^ T cells^1^,^24^, we quantified tetramer staining within the CD4-negative population. Frequencies of MR1-Ag tetramer^+^ cells ranged from 0.98 to 4.30% (mean 2.30%, *n*=5) of the CD4-negative lymphocytes (Fig. 6, top row). In line with previously published data^10^, the majority of MR1-Ag tetramer^+^ cells expressed the TRAV1-2 TCR (Fig. 6, second row). However, on average, 2.57% of MR1-Ag tetramer^+^ cells did not express TRAV1-2 (red events in Fig. 6). Furthermore, these were present in all donors and ranged in frequency from 1.40 to 4.22% of tetramer^+^ cells. TRAV1-2^+^ MAIT cells have been phenotypically characterized as cells with high expression of CD161 (refs 26, 38) and CD26 (refs 7, 30). In line with this, the majority of the TRAV1-2^+^ MR1-Ag tetramer^+^ cells co-expressed CD26 and CD161 (mean 96.2%, s.d. 2.5%; Fig. 6, bottom row). In contrast, a smaller proportion of tetramer^+^ TRAV1-2-negative cells expressed CD161 and CD26, although there was considerable heterogeneity between donors (mean 60.1%, s.d. 21.6%, range 37.8–86.2%). To exclude the possibility that tetramer binding masked the staining of TRAV1-2, we depleted the TRAV1-2^+^ cells using FACS in three of the above donors, and then stained the remaining TRAV1-2-negative cells with the MR1 tetramer. In this case, the estimated TRAV1-2-negative tetramer frequencies were nearly identical to those seen before TRAV1-2 depletion (Fig. 6, bottom). This experiment verified the frequencies of TRAV1-2-MR1-restricted T cells from each donor. In sum, these data confirm the presence of T cells capable of interacting with MR1-Ag that do not express the TRAV1-2 TCRα in healthy human blood.

Finally, in order to test the generalizability of the antigenic reactivity observed by clone D462-E4, we generated CD8^+^ TRAV1-2-negative MR1 tetramer^+^ T-cell lines from the four additional donors used in Fig. 6. Importantly, these T-cell lines were enriched to 84–97% TRAV1-2-negative of MR1 tetramer^+^ cells. We observed equivalent MR1-restricted IFN-γ reactivity to *M. smegmatis* and *S. pyogenes* infection by each T-cell line. Taken together, the confirmation of our finding from four additional PBMC donors clearly supports generalized reactivity of TRAV1-2-negative MR1-restricted T cells across individuals.

## Discussion

While MAIT cells have been defined through their usage of the TRAV1-2 TCR, in this report we demonstrate unambiguously the presence of MR1-restricted T cells that are TRAV1-2-negative, demonstrate the specific usage of the TRAV12-2 TCR by a clone and find that these cells are capable of recognizing both the previously demonstrated RL riboflavin intermediates, as well as unique ligands derived from *S. pyogenes*, a bacterium incapable of riboflavin biosynthesis. As a result, our study demonstrates considerable promiscuity in MR1-restricted T cells, at the level of the ability of their TCR to recognize antigens, and in the ability of MR1 to present these ligands.

Here we find that TRAV12-2 MR1-restricted T cells can be stained with the MR1-Ag tetramer, and have the ability to recognize both known RL antigens as well as an antigen derived from *S. pyogenes*. The observation that the TRAV1-2 MR1-restricted T cell cannot recognize this bacterium provides definitive evidence that the antigen being recognized is distinct. These data, then, would suggest a model in which the MAIT cell TCR confers selectivity, but not stringent specificity. Similarly, our data support the hypothesis that MR1 is capable of presenting an array of ligands. The observation that MR1-restricted T cells of varying TCR usage and antigenic selectivity can be broadly defined by staining with the MR1-Ag (5-OP-RU) tetramer is reminiscent of CD1d-restricted T cells defined by staining with the αGalCer tetramer^39^,^40^,^41^. In these initial studies, human NKT cells were enumerated using CD1d-αGalCer tetramers and found to stain with anti-Vα24 TCR antibody. More recently, populations of NKT cells expressing alternative semi-invariant TCRs that bind to αGalCer-loaded CD1d tetramers have been identified^42^,^43^. Elucidation of the crystal structure of one of these alternative TCRs to αGalCer-loaded tetramer showed a similar binding mode to that of the Vα24Jα18 TCR^44^. Taken together, this shared phenomenon between MR1 and CD1d antigen presentation must be what allows selective activation within the confines of microbial pattern recognition by unconventional T cells.

The use of TRAV12-2 TCR by MR1-restricted T cells necessarily challenges the existing paradigm of how the MAIT cell TCR interacts with MR1. Prior studies have defined the structural and functional requirements of the semi-invariant TRAV1-2 TCR for MAIT cell activation in the context of MR1 and bound ligand^4^,^18^,^19^,^20^,^21^. These studies define a clear role for the CDR3α and possibly the CDR3β loop in ligand recognition. Specifically, these studies suggested a conservation of the key patterns of TCR residues in the TCR α-chain including the conserved amino acid, Tyrα95 (refs 18, 19, 20, 21, 22, 45). The critical tyrosine at position 95 in the CDR3 of the TCRα chain allows for the formation of a hydrogen bond with MAIT activating but not non-activating ligands. For example, MR1 binding of the RL-6-Me-7-OH ligand (that is recognized by D462-E4) allows for a single TRAV1-2^+^ TCR contact with TRAV1-2: Tyr95 of the CDR3α loop^20^. While this residue is highly conserved between MAIT TCRs^25^,^46^, sequence analysis of TRAV12-2^+^ D462-E4 demonstrates that this clone lacks a tyrosine residue in its CDR3α region. We note that we have previously reported that a proportion of microbial-reactive, TRAV1-2-expressing MR1-restricted T cells do not contain the Tyrα95 (ref. 23). A recent study by Gherardin *et al.*^28^ observed that a TRAV1-2-negative MR1-restricted TCR (TRAV36/TRAJ34) could instead use an asparagine residue of its CDR1α to contact the 5-OP-RU-activating ligand. This elegant study highlights that alternative molecular interactions can mediate atypical TCR recognition of MR1-Ag. At present, we cannot comment on the critical residues that mediate the D462-E4 TCR interaction with MR1 ligand.

Our data clearly support the hypothesis that MR1 can present a diverse array of ligands to MR1-restricted T cells. First, by comparing microbial recognition of the TRAV1-2 and TRAV12-2 TCR we have defined patterns of recognition that would imply the presence of more than one activating ligand. For instance, the ability of TRAV1-2-expressing T cells to uniquely recognize a microbe would suggest the presence of a ligand not recognized by TRAV12-2 TCR. Similarly, those microbes that are preferentially recognized by the TRAV1-2 T cells likely contain either a single ligand that is recognized preferentially (analogous to our findings of ligand discrimination between RLs in Fig. 4b), or containing multiple ligands. Most striking, however, was the ability of the TRAV12-2^+^ MR1-restricted T cell to recognize *S. pyogenes*, an organism that cannot synthesize riboflavin. Because this pathogen is not recognized by the TRAV1-2^+^ T cells, these data would unambiguously refute the hypothesis that differential MAIT cell recognition can be simply explained by differing proportions of riboflavin metabolites. At present, the MR1 ligand from *S. pyogenes* remains to be determined. Recent molecular analyses suggest that MR1 can accommodate a range of different ligands because of plasticity in ligand orientation of the binding cavity^4^,^18^,^19^,^20^,^21^,^22^. As the pterin ring occurs commonly in the environment, it is feasible that other microbial or host molecules with common chemotypic properties could bind to MR1, and function as antigens for MR1-restricted T cells. We hypothesize that diversity in MR1 ligands allows MR1-restricted T cells to recognize a wide range of microorganisms and their associated metabolomes.

In contrast to conventional T-cell populations, MAIT cells can be found in the thymus with effector pathogen-reactive capability^13^ and their selection depends on haematopoietic rather than epithelial cells^12^,^38^,^47^. It is unknown whether innate T-cell function is a T-cell-intrinsic programme or is a result of TCR signalling through selection in the thymus by MR1. Both functional data and tetramer staining demonstrate the presence of MR1-restricted T cells in all donors. Because we do not have a TRAV12-2 antibody, the full TCR repertoire of these cells remains to be determined as well as whether they share the innate T-cell attributes and selection pathway of TRAV1-2+ MR1-restricted T cells.

We also note the preponderance of MR1-restricted T cells expressing the TRAV1-2 TCR, in line with prior observations^10^. This phenomenon could occur by a scenario where TRAV1-2^+^ T-cell selection in the thymus is favoured over other TCRs. Alternatively, given evidence of ligand discrimination by MAIT TCRs, TRAV1-2^+^ MAIT cells could dominate in the periphery because of selective microbial exposures. In line with this hypothesis, perhaps repeated exposures of environmental mycobacteria or Gram-negative gut microbiota allow for preferential expansion of TRAV1-2^+^ MR1-restricted T cells in the majority of individuals.

On the basis of our findings, we propose that non-TRAV1-2 MR1-restricted TCRs contribute to immune defence against infection primarily by providing more diverse and, in some instances, unique microbial recognition. For instance, the TRAV12-2^+^ MR1-restricted T-cell clone can recognize infection with *S. pyogenes*. A variety of diseases are caused by infection by *S. pyogenes* or Group A streptococcus^48^. These include throat infection ‘strep throat', pneumonia, fasciitis, nosocomial wound infection and glomerulonephritis. We hypothesize that MR1-restricted T cells expressing TRAV12-2^+^ or other atypical TCRs selectively expand at tissue sites, such as the human mouth, tonsils and skin, associated with streptococcal infection. Our prior finding of microbe-selective clonal MR1-restricted T-cell expansions within individuals^23^, in conjunction with the data presented herein, demonstrates the capacity of MR1-restricted T cells to discriminate between microbial infections, and supports the hypothesis that MAIT cells display antigen-driven clonal expansion.

In sum, we show that MR1-restricted T cells have the capacity to detect a greater diversity of microbes than previously shown. We have isolated a human T-cell clone that expresses a TCR never observed within MAIT cells before, TRAV12-2, demonstrating that MR1-restricted T cells do not use TRAV1-2 exclusively. This TRAV12-2 TCR displays MR1-Ag discrimination both with regard to the recognition of known RL metabolites, and most notably in its capacity to uniquely detect *S. pyogenes*, a pathogen that lacks the capacity to synthesize riboflavin. Collectively, these data provide evidence that additional MAIT cell subsets may play a unique role in human defence against infection by broadening the recognition of microbes and their associated metabolites.

## Methods

### Human participants

All samples were collected and all experiments were conducted under protocols approved by the institutional review board at Oregon Health and Science University. PBMCs were obtained by apheresis from healthy adult donors with informed consent.

### Cell lines and reagents

All cell lines used in this study have been confirmed to be mycoplasma-free. The A549 lung carcinoma cell line (ATCC CCL-185) was used as APCs for IFN-γ ELISPOT assays in Figs 1 and 2, direct *ex vivo* intracellular cytokine staining determination of microbe-reactive T cells and in Fig. 4c for infection with *Neisseria gonorrhoeae* and *Y. enterolitica*. The BEAS2B bronchial epithelial cell line was used (ATCC CRL-9609) in Fig. 3 for antibody blockade ELISPOT assays. Cell lines were maintained by continuous passage in F12K culture medium supplemented with 10% fetal bovine serum. RL-6,7-diMe and RL-6-Me-7-OH were purchased from WuXi Apptec and 6FP from Schick Laboratories. Live-dead aqua stain and carboxyfluorescein succinimidyl ester (CFSE) were purchased from Life Technologies. Unconjugated antibodies used in the study were the following: anti (α)-CD3 (clone OKT3), αβTCR (clone T10B9, BD), αMR1 (26.5, gift from Ted Hansen), αHLA-ABC (W6-32, AbD Serotec), αCD1a/CD1b/CD1c/CD1d (gift from Branch Moody), αIFNγ for ELISPOT (Mabtech, see ELISPOT methods section below) and LEAF purified IgG2a, IgG1 and IgM isotype controls (Biolegend). Conjugated antibodies used in this study were the following: αCD3 (UCHT1, fluorescein isothiocyanate (FITC) or PerCP Cy5.5, 1:50 dilution, Biolegend, BL), αTRBV29-1 FITC (1:10 dilution, Beckman Coulter), αCD8α (APC-Cy7, 1:500 dilution, clone RPA-T8, BL), αCD26 (FITC, 1:50 dilution, clone BA5b, BL), αCD161 (PE-Cy7, 1:50 dilution, HP-3G10, BL), αCD4 (Brilliant violet 785, 1:50 dilution, OKT4, BL), αIFNγ FITC (1:25 dilution, Beckman Coulter), αβTCR (phycoerythrin (PE), 1:50 dilution, clone T10B9, BD) and IgG2a isotype conjugated to FITC (BD), αTRAV1-2 (APC, 1:50 dilution, OF5A12)^13^, αCD247 pY142 (PE, 1:10 dilution, BD Phosflow), αZAP-70 pY319/Syk pY352 (PE, 1:10 dilution, BD Phosflow) and αTCRγδ (FITC, 1:25 dilution, clone B1, BD). 5-OP-RU: 5-(2-oxopropylideneamino)-6-D-ribitylaminouracil.

### Monocyte-derived DCs

PBMCs obtained by apheresis were resuspended in 2% human serum (HS) in RPMI and were allowed to adhere to a T-75 flask at 37 °C for 1 h. After gentle washing twice with PBS, nonadherent cells were removed and 10% HS in RPMI containing 30 ng ml^−1^ of IL-4 (Immunex) and 30 ng ml^−1^ of granulocyte–macrophage colony-stimulating factor (Immunex) was added to the adherent cells. The cells were X-rayed with 3,000 cGray using X-RAD320 (Precision X-Ray Inc.) to prevent cell division. After 5 days, cells were harvested with cell-dissociation medium (Sigma-Aldrich, Gillingham, UK) and used as APCs in assays.

### Generation of an MR1-knockout cell line using CRISPR/Cas9

The reagents used to mutate the MR1 gene were derived from the toolkit described in ref. 49. A codon-optimized synthetic Cas9 cDNA under the control of the cytomegalovirus promoter (Addgene plasmid #41815) was used in combination with a single guide RNA comprising a transactivating CRISPR RNA sequence^49^ as well as the 19-nucleotide protospacer sequence (5′-GATGGGATCCGAAACGCCC-3′) targeting the + strand of exon 3 of the *MR1* gene followed by the protospacer-associated motif AGG. Plasmid DNA serving as template for the transcription of the CRISPR/Cas9 elements was transfected in the carcinomic human alveolar basal epithelial cell line A549 using Lipofectamine 2000 (Invitrogen, Life Technologies, Paisley, UK) according to the manufacturer's instructions. Genomic DNA from A549 cells was isolated with the GenELute mammalian genomic DNA miniprep kit (Sigma-Aldrich). Mutations at the target site were detected using the CEL-I enzyme, as part of the SURVEYOR assay (Transgenomic Ltd, Glasgow, UK), which cleaves DNA duplexes bearing base pair mismatches, caused by insertions or deletions at proximity of the protospacer-associated motif sequence, within the PCR amplicons generated with primers flanking the genomic target site. The PCR forward primer (5′-GCATGTGTTTGTGTGCCTGT-3′) is located in the intron region upstream of the target site and the reverse primer (5′-GGTGCAATTCAGCATCCGC-3′) downstream on exon 3. MR1 protein expression at the cell surface was measured using flow cytometry with the anti-MR1 antibody clone 26.5 (a kind gift from Professor Ted Hansen) following overnight stabilization by incubating cells with 50 μg ml^−1^ acetyl-6-formylpterin (Schirks Laboratories, Jona, Switzerland). MR1-negative cells were sorted using flow cytometry and single-cell clones were derived from the sorted bulk population by limiting dilution (average of 0.3 cells per well). Clonal populations were then screened for MR1 surface expression and DNA indels with the SURVEYOR assay. The region flanking the target site was PCR-amplified from the genomic DNA of selected clones using the primers described above fused to restriction sites and the PCR products were cloned into recipient plasmids, which were transfected in chemically competent Top10 *E. coli* bacteria. Ten colonies that tested positive for the insert by colony PCR were used to produce plasmid Minipreps, which were sent for Sanger sequencing in order to determine the nature of CRISPR/Cas9-induced mutations. Clone 9 (in the manuscript as MR1^−/−^) was shown to bear a 125 bp deletion on one allele and a single bp deletion on the other.

### Isolation of TRAV1-2-negative T-cell lines and clones

CD8+, CD4−, γδ TCR− and TRAV1-2-negative T cells were FACS-sorted from PBMCs, and 1 × 10^6^ were added to 1 × 10^5^ irradiated (3,500 rad using a Cs source) monocyte-derived autologous DCs infected with *M. smegmatis* (MOI 3) and incubated in RPMI 1640 with 10% HS, rhIL-2 (2 ng ml^−1^) and rhIL-12 (0.5 ng ml^−1^). Seven days later, T cells were removed and stained with CFSE, and then added to 1 × 10^5^ irradiated (3,500 rad) monocyte-derived autologous macrophages infected with *M. smegmatis* (MOI 3) and incubated with rhIL-2 (2 ng ml^−1^) for another week. The resulting T-cell line was FACS-sorted for CFSE-diluted cells. The sorted T cells were rested overnight in RPMI 1640 supplemented with 10% HS and 0.5 ng ml^−1^ rhIL-2. The CFSE-sorted T-cell line was rapidly expanded using anti-CD3 following the protocol written below, then FACS-purified based on its ability to bind MR1 tetramer loaded with 5-OP-RU (ref. 4), rested overnight with rhIL-2 (0.5 ng ml^−1^) and rapidly expanded using anti-CD3 again to generate ‘D462-E4' T-cell clone.

### Expansion of T-cell clones

T-cell clones were cultured in the presence of X-rayed (3,000 cGray using X-RAD320, Precision X-Ray Inc.) allogeneic PBMCs, X-rayed allogeneic LCL (6,000 cGray) and anti-CD3 monoclonal antibody (20 ng ml^−1^; Orthoclone OKT3, eBioscience) in RPMI 1640 media with 10% HS in a T-25 upright flask in a total volume of 30 ml. The cultures were supplemented with IL-2 on days 1, 4, 7 and 10 of culture. The cell cultures were washed on day 5 to remove soluble anti-CD3 monoclonal antibody.

### Phosphorylation-specific T-cell staining for flow cytometry

T-cell clones were incubated overnight in RPMI media containing 0.5 ng ml^−1^ IL-2 and 2% HS. Monocyte-derived DCs were incubated for one hour with *S. pyogenes* or *M. smegmatis* MOI=10, or nothing, in ultra low adherence culture plates. A three to one ratio of DCs to T cells was co-incubated for 15 min at 37 °C and then immediately fixed in 2% paraformaldehyde. Following fixation, the cells were permeabilized in ice-cold 100% methanol for 30 min. Then, the cells were washed in FACS buffer to sufficiently remove the methanol and stained with the following antibodies: anti-CD8 (clone RPA-T8, 1:50 dilution, Biolegend), anti-CD3ζ-pY142 (1:10 dilution, BD), anti-ZAP-70 pY319/Syk pY352 (1:10 dilution BD) or isotype controls (IgG2a for anti-CD3ζ, IgG1 for anti-ZAP-70, used at a matching concentration to their orresponding phospho-specific antibody) for 45 min at 4 °C. Isotype controls were used to optimize staining with phospho-specific antibodies. We use the mitogen PHA as a positive control for TCR stimulation and maximum phosphorylation signal. A minimum of 30,000 CD8^+^ events were collected for geometric mean fluorescence intensity analysis across samples.

### Analysis of TCR usage

Amplification and sequencing of TCRB and TCRAD CDR3 regions were performed using the immunoSEQ Platform (Adaptive Biotechnologies, Seattle, WA). ImmunoSEQ combines multiplex PCR with high-throughput sequencing and a bioinformatics pipeline for (TCRB/TCRAD) CDR3 region analysis^50^,^51^. The IMTG nomenclature was used throughout the study^52^.

### Flow cytometry staining and cell sorting

Cells to be analysed for cell surface marker expression were first incubated at 4 °C in a blocking solution of PBS containing 2% normal rabbit serum (Sigma-Aldrich), 2% normal goat serum (Sigma-Aldrich) and 2% HS to prevent nonspecific binding. Cells were washed in PBS and then incubated with live-dead discriminator and surface stains or isotype controls for 20 min in the dark at 4 °C in a total volume of 50 μl. Cells were then washed and fixed, or fixed and permeabilized (*ex vivo* ICS tests, BD Fix/Perm kit) according to the manufacturer's instructions. Antibodies for intracellular staining were then added for 30 min in the dark at 4 °C in a total volume of 50 μl, and after washing flow cytometry was performed. Specifically for Fig. 3a, 2 × 10^6^ PBMCs from each donor were stained with the MR1-Ag tetramer at 0.3 nM in 25 μl volume for 45 min in PBS buffer containing 2% fetal bovine serum at room temperature in the dark. Viability and surface stains were added on top of the tetramer stain for another 20 min at 4 °C. Samples were then washed twice in tetramer staining buffer. All flow cytometry analyses were performed on a Fortessa 18-parameter flow cytometer (BD). All FACS analyses were performed using an Influx 11-parameter flow cytometer (BD) with the Oregon Health and Science University flow cytometry core facility. Data were analysed using FlowJo (v9.8.5). Fluorescence minus one controls were used for optimal gating. Doublets were excluded based on FSC-H and FSC-A, SSC-H and SSC-A; lymphocytes were identified based on FSC-A and SSC-A and CD3 expression; and dead cells were excluded based on Aqua viability dye.

### *Ex vivo* stimulation assay

To observe the nonclassical T-cell response, we used the A549 cell line as APCs because it does not express MHC-II and is MHC-I-mismatched to the donor source of T cells. CD8+ T cells were positively selected from healthy donor PBMCs using magnetic bead separation according to the manufacturer's instructions (Miltenyi), added to uninfected WT or *M. smegmatis*-infected (MOI=3, overnight infection) WT or MR1^−/−^ A549 cells at a ratio of 3:1 and incubated overnight in the presence of Brefeldin A and 0.5 ng ml^−1^ rhIL-2 at 37 °C. The following day, the cells were stained for surface phenotype markers and live-dead discriminator. Following surface staining, cells were fixed and permeabilized using the BD Fix/Perm Kit and stained intracellularly with α-IFN-γ.

### Microorganisms and preparation of APCs

*M. smegmatis, C. albicans, S. enterica Typhimurium, E. coli, M. tuberculosis, S. pyogenes* and *M. avium* were used from frozen glycerol stocks, whereas all other microbes were harvested from overnight growth on agar plates and titred based on OD_600_ readings of a colony suspension. A cell-free supernatant was created from an overnight culture of *S. pyogenes* (ATCC 19615) that was sterile-filtered and frozen before being used in T-cell stimulation assays of Fig. 5c–f. A549 cells were infected for 2 h or DCs were infected for 1 h with microbes at 37 °C. In fig. 4c, A549 cells were used for Yersinia and Shigella infections; DCs were used for all other infections. The MOI and antibiotics used for each microbe were optimized for APC viability and maximal MR1-restricted response: *E. coli* 1, *M. smegmatis* 3, *S. flexneri* 1, *Y. enterolitica* 1, *C. albicans* 0.1, *M. avium* 30, *N. asteroides* 1, *S. enterica* Typhimurium 30, *V. parahemolitica* 1, *M. tuberculosis* 30, *N. gonorrhoeae* 1, *P. aeruginosa* 1, *S. aureus* 1, *S. pyogenes* 30, *E. faecalis* 1 and 10. All infections were performed in the absence of antibiotics. After the indicated infection time, all cells were washed twice in media containing antibiotics and then incubated overnight in an ultra low adherence tissue culture plate before being counted and added to the assay (ELISPOT set-up described below).

### Riboflavin dependence growth assay

Overall, 5 × 10^6^ colony-forming unit from frozen glycerol stocks of *S. pyogenes* or *E. coli* were added to 10 ml of minimal growth media and cultured at 37 °C for 96 h (*S. pyogenes*) or 12 h (*E. coli*) in the dark. Minimal growth media was made with M9 salts (BD Difco) supplemented with glucose, MgSO_4_ and CaCl_2_, as recommended by the manufacturer, and 0.01% w/v amino acids (casein enzymatic digest, Sigma). Riboflavin (Sigma) was added to the growth medium at μM concentrations indicated in Fig. 5. Bacterial growth was measured by absorbance readings at 600 nm.

### IFN-γ ELISPOT assay and antibody blocking

A MSHA S4510 96-well nitrocellulose-backed plates (Millipore, bought via Fisher Scientific) was coated overnight at 4 °C with 10 μg ml^−1^ solution of anti-IFN-γ monoclonal antibody (Mabtech clone 1-D1K) in a buffer solution of 0.1 M Na_2_CO_3_, 0.1 M NaHCO_3_, pH=9.6). Then, the plate was washed three times with sterile PBS and blocked for 1 h at room temperature with RPMI 1640 media containing 10% heat-inactivated HS pool. Then, the APCs and T cells were prepared as described below and co-incubated overnight. Briefly, DCs (Figs 4a,d and 5), the BEAS2B cell line (Fig. 3d) or the A549 cell line (all other experiments) were used as APCs at 1 × 10^4^ per well in ELISPOT assays. For all blocking ELISPOT assays, APCs were limited to 5 × 10^3^ per well. In Fig. 4b,c, the A549 cell line was incubated with MAIT antigens over a range of concentrations in the dark for 2 h. Where stated, blocking antibodies or antagonists were added for 2 h at 2.5 μg ml^−1^ (α-HLA-I clone W6/32, α-CD1a, b, c, d (Branch Moody), 6-formyl pterin (50 μg ml^−1^, Schick Laboratories) and α-MR1 clone 26.5 (Ted Hansen) or appropriate isotype controls). To block the T-cell receptor, anti-αβTCR (clone T10B9 (ref. 53), BD Pharmingen) or isotype control was added to T-cell clones at 0.5 μg ml^−1^ for 30 min at 4 °C before co-incubation with APCs, T-cell clones were added at 1 × 10^4^ per well. The plate was incubated overnight at 37 °C and then washed six times with PBS containing 0.05% Tween. The plate was then incubated for 2 h at room temperature with a 1 μg ml^−1^ solution of anti-IFN-γ-biotin secondary antibody (Mabtech clone 7-B6-1) in 0.5% BSA, 0.05% Tween in PBS. Finally, the plate was washed six times in PBS-Tween, and then PBS, and developed using an AEC Vectastain kit SK-4200 (Vector labs). We defined a positive response as greater than 25 IFN-γ spot-forming units.

### Preparation of pulsed fixed APC

Monocyte-derived DCs were pulsed for 4 h with bacterial culture supernatant from *S. pyogenes* or left untreated and then washed and rested overnight in an ultra low adherence tissue culture plate. The following day, the pulsed DC-conditioned media (Fig. 5c) was collected and added to the D462-E4 clone in the ELISPOT plate. Then, half of each harvested DC sample was used in the ELISPOT as ‘unfixed'. The other half was fixed by incubating in 0.5% paraformaldehyde (Electron Microscopy Sciences) in PBS for 15 min at room temperature. Then, an equal volume of 0.4 M lysine was added to stop the reaction for 5 min. The samples were then extensively washed with media, incubated for 1 h at 37 °C and then washed again before being used in the ELISPOT assay.

### Data Analysis

Flow cytometry data were analysed using FlowJo software 9.8.1 (Tree Star). All statistical analyses were performed using the Prism software with nonparametric, the Mann–Whitney *U*-tests (Fig. 2c). Nonparametric statistical tests were used for small group sizes (five donors in this study). In all descriptive statistical analyses, the variance was first confirmed to be similar between groups using s.d. or s.e.m. tests, as appropriate and displayed on each graph. *P* values≤0.05 were considered significant (*P≤0.05).

### Data Availability

The authors declare that the data supporting the findings of this study are available within the article and its Supplementary Information Files, or are available from the corresponding author upon request.

## Additional information

**How to cite this article**: Meermeier, E. W. *et al.* Human TRAV1-2-negative MR1-restricted T cells detect *S. pyogenes* and alternatives to MAIT riboflavin-based antigens. *Nat. Commun.* 7:12506 doi: 10.1038/ncomms12506 (2016).

## Supplementary Material

Supplementary Information Supplementary Figures 1-2 and Supplementary Table 1.
