# Supplementary material for: Human TRAV1-2-negative MR1-restricted T cells detect S. pyogenes and alternatives to MAIT riboflavin-based antigens
Source: Nat Commun. 2016 Aug 16;7:12506. doi: 10.1038/ncomms12506 (PMC4990709; doi:10.1038/ncomms12506)
Supplement: Supplementary Information — Supplementary Figures 1-2 and Supplementary Table 1. [file ncomms12506-s1.pdf]

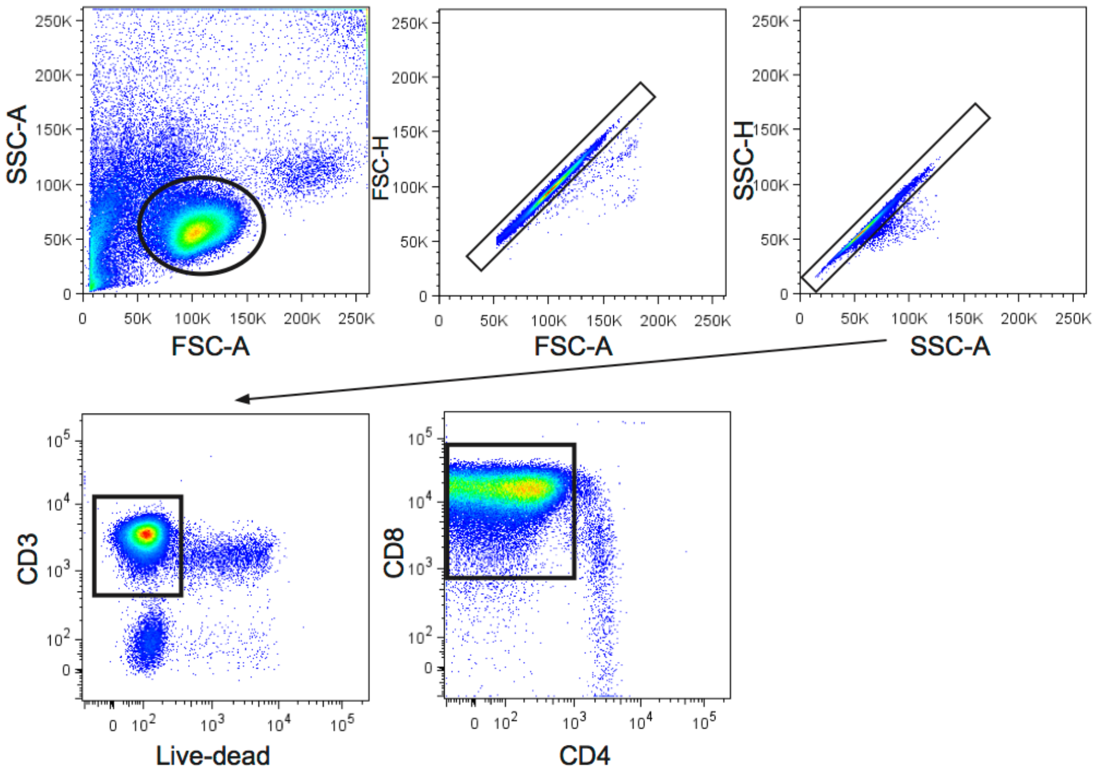

Supplementary Figure 1. Flow cytometry gating strategy for the $CD8^+$ PBMC T cell stimulation assay.

All events were first gated on a uniform cell size using area parameters and then for single events using area versus height parameters. Then T cells were selected for expression of CD3 and exclusion of a dye indicating their viability (Live-dead). Finally, T cells that expressed CD8, but not CD4, were analyzed for their intracellular cytokine expression.

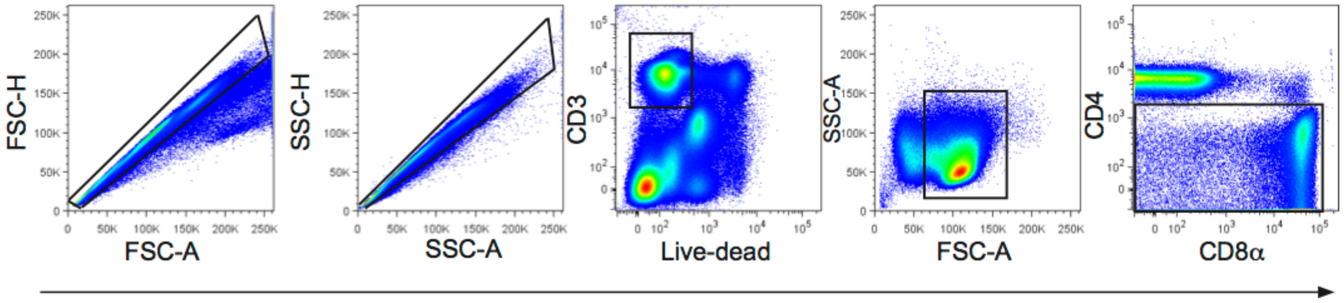

Supplementary Figure 2. Flow cytometry gating strategy for MR1/Ag tetramer staining.

All events were first gated on single cell events using area and height parameters. Then cells were selected for expression of CD3 and exclusion of a dye indicating their viability (Live-dead). Next, cells were gated on a uniform lymphocyte size. Finally, T cells that did not express CD4 were analyzed for their ability to bind the MR1 tetramer.

Supplementary Table 1. Nucleotide sequences of the junctional regions surrounding the CDR3 and T cell receptor of D462-E4

Nucleotide sequence	CDR3 Amino Acid	CDR3 Length	V Gene Name	J Gene Name
CATCAGAGACTCCCAGCCCAGTGATTCAGCCACCTACCTCTGTGCCGTGAGAGATGCAGGCAACATGCTCACCTTTGGAGGG	CAVRDAGNMLTF	36	TCRAV12-02	TCRAJ39-01
AACATGAGCCCTGAAGACAGCAGCATATATCTCTGCAGCGTGGGGGGGGACAGCCTTATAGGCAATCAGCCCCAGCATTTTGGTGAT	CSVGGDSLIGNQPQHF	48	TCRBV29-01	TCRBJ01-05
